# Do conspiracy theory and mistrust undermine people's intention to receive the COVID‐19 vaccine in Austria?

**DOI:** 10.1002/jcop.22714

**Published:** 2021-09-22

**Authors:** Phil Knobel, Xiang Zhao, Katherine M. White

**Affiliations:** ^1^ Institute of Psychology University of Klagenfurt Klagenfurt am Wörthersee Austria; ^2^ School of Law, Psychology and Social Work Örebro University Örebro Sweden; ^3^ School of Psychology and Counselling Queensland University of Technology Brisbane Australia

**Keywords:** attitude, conspiracy, COVID‐19, intention, vaccination

## Abstract

Conspiracy theories flourish during the coronavirus disease 2019 (COVID‐19) pandemic especially regarding vaccinations. As the vaccination reluctancy in Austria is high, it is important to understand the antecedents of vaccination intention at the preapproval stage of the vaccination process. An online survey was conducted in August 2020 in Austria with 217 primarily younger, female, educated participants. A two‐step cluster analysis resulted in a sceptics cluster with a clear antivaccination tendency along with a right‐wing political position, lower trust in general vaccines and lower education levels and the reference cluster. A considerable percentage of participants reported their reluctancy to have a COVID‐19 vaccine. Although vaccination intention can be explained by attitude and subjective norm, this decision‐making process is undermined by underlying factors such as conspiracy ideation and political position. Policy makers and health interventionists should take political background into consideration in efforts to increase vaccine compliance.

## 1 INTRODUCTION

### 1.1 COVID‐19 and its vaccines

Since the outbreak of the novel coronavirus disease (COVID‐19) by the end of 2019, a worldwide pandemic subsequently developed (Hufert & Spiegel, 2020). This disease leads to serious health implications which are thus far not fully understood (Guan et al., 2020; Rawson et al., 2020; Wadman et al., 2020; Weiss & Murdoch, 2020). The pandemic has negatively affected societal structure and everyone's life (Oxford Analytica, 2020).

As no effective treatment for COVID‐19 has been found yet, this new normality (i.e., measures including lockdowns, curfews, flights restrictions) will continue until a safe vaccine has been developed and implemented which will raise immunity at the individual and population level. In December 2020, 44 different vaccines against COVID‐19 are in clinical trials for their efficacy and safety (Haque & Pant, 2020). Some of the vaccines are close to final approval in the United States and the European Union (Haque & Pant, 2020), while the UK approved two vaccines in early December (Mahase, 2020) and started vaccinating their population. Noticeably, Russia has approved a COVID‐19 vaccine in August 2020 (“Sputnik V”, a polically infused name after an artificial satellite launched by the Soviet Union), without Phase 3 trials (Burki, 2020).

### 1.2 Intention to be vaccinated

From an individual perspective, one's decision to get vaccinated is a voluntary action, and thus can be regarded as a volitional behaviour as one's subjective evaluations of vaccines and perceived social pressure to be vaccinated both could leverage one's intention to get vaccinated (Myers & Goodwin, 2011; Schmid et al., 2017). One's vaccination intention is influenced by limited knowledge and active resistance against the health recommendations (Bangerter, 2014). As negative attitudes toward a vaccine serve as a major barrier to vaccinations (Schmid et al., 2017), it is important to assess attitudes toward impending COVID‐19 vaccines. Such studies would help society members to build trust and vaccination readiness, especially urgent for European countries where the world's lowest confidence in vaccine safety is found (Larson et al., 2016).

To understand one's intention to take the COVID‐19 vaccine, we opted for the Theory of Reasoned Action (TRA; Fishbein & Ajzen, 1975). The TRA is a plausible theoretical framework to explain people's intention to a specific behaviour. According to the TRA, one's intention to take the COVID‐19 vaccine is determined by both attitude (i.e., positive or negative opinions about the COVID‐19 vaccine) and subjective norm (i.e., perceived social pressure to have the COVID‐19 vaccine) (Fishbein & Ajzen, 1975). Bearing in mind that there are several extended models of the TRA (e.g., Ajzen's (1991) Theory of Planned Behaviour, which also incorporates one's perceived behavioural control), we choose the TRA because our focus was the preimplementation stage of vaccine approval. This stage is of uttermost importance for the genesis of individuals' beliefs and intentions toward the vaccine, assisting in understanding people's preliminary perspectives and signalling likely vaccine uptake. Given during this preimplementation there is an absence of control‐related specifics such as price, availability and dosage, we assumed that people had insufficient information to assess the degree to which it is under their control to have the vaccine.

### 1.3 Conspiracy theories

Pandemics usually come with a great uncertainty and mistrust against authorities and medical experts (Taylor, 2019); for example, the 1832 cholera outbreak in Liverpool (Burrell & Gill, 2005), the novel influenza A (H1N1) pandemic in 2009 (Smallman, 2015), and the Ebola epidemic in African countries (Nguyen, 2019). Unofficial knowledge and negative emotions such as fear that are often perceived in pandemics may both breed conspiracy theories (van Prooijen, 2018).

People who believe in conspiracy theories during public health challenges may know less about the disease, or have more mistrust against medical organizations, or hold more xenophobic stereotypes (Earnshaw et al., 2019). When fake news and conspiracy theories are regarded as credible information, people may show less trust in the government and medical organizations (Ognyanova et al., 2020). In contrast, political trust (i.e., one's assessment of the core institutions of the polity; Zmerli, 2014) is protective against the beliefs in conspiracy ideation, which gives policy makers the possibility to increase community trust to reduce conspiracy ideation (Goldberg & Richey, 2020). While more research is needed to understand the development of public trust in health research fields (Larson et al., 2011), timely studies examining COVID‐19 vaccines are limited.

During the COVID‐19 pandemic, research found that believing more conspiracy beliefs were associated with the refusal of vaccination and support for hydroxychloroquine (Bertin et al., 2020). A multinational study with data from 5000 participants from the United States, the UK, Ireland, Mexico, and Spain clearly shows the link between susceptibility and misinformation, vaccine hesitancy, and reduced compliance with public health measures during the COVID‐19 pandemic (Roozenbeek et al., 2020). Higher vaccination refusal among people with conspiracy inclination was also found in a recent study in the United States (Romer & Jamieson, 2020). While conspirators tend to show stronger antivaccination beliefs and political mistrust (Goldberg & Richey, 2020), their reasoning patterns are usually difficult to change (Taylor, 2019).

### 1.4 COVID‐19 in Austrian context

European countries have the world's lowest confidence in vaccine safety (Larson et al., 2016), partly generating immunological naivety as well as herd immunity strategies and subsequently putting people at risk at a societal level. Previous local outbreaks of diseases such as measles that could have been mitigated via higher vaccination adherence had mirrored this mindset (Datta et al., 2018). Our current study focuses on a central European country: Austria.

After the first two COVID‐19 cases in Austria were detected in late February 2020, the daily new cases increased to around 1000 a few weeks later. A lockdown was implemented at the end of March as a containment strategy. After a calm phase over the summer, the second wave started around mid‐August, peaking in November when almost 10,000 daily new cases were reported. After a second lockdown, new infected cases were showing a declining trend, but such figures were quite high for a country with approximately nine million inhabitants. While Austrians seem to be satisfied with the official interventions during the COVID‐19 pandemic (Willems et al., 2020), 20%–40% of the general population believed the danger of COVID‐19 had been exaggerated (Gallup Institut, 2020a).

Historically, Austrians do not show a strong adherence to vaccination. For example, only 70% of the population underwent the complete measles vaccination procedure, with increasing numbers of nonvaccinated children and youths (Brettenhofer et al., 2019; Rosian & Habl, 2003). For the impending COVID‐19 vaccines, a survey in mid‐May found that 62% of Austrians were open to the vaccine for COVID‐19; this figure reflected much higher rates than the seasonal influenza vaccination (Austria Presse Agentur, 2020). Curiously, this willingness fluctuated from month to month. One study noted while in May 48.2% were provaccination, in October only 33.8% agreed to be vaccinated (Kittel, 2020). Notably, most Austrians disagreed with a mandatory vaccine for COVID‐19 (Eberl et al., 2020; Gallup Institut, 2020b).

Reasons for Austrians' vaccination hesitancy include the fear of side effects and mistrust in the protective effects of a vaccination (Gallup Institut, 2020b). Interestingly, vaccination hesitancy among Austrians also seems to be related to the support of Austria's right‐wing populist party (FPÖ/Freiheitliche Partei Österreichs), lower perceived threat of COVID‐19, as well as stronger conspiracy ideation (Eberl et al., 2020).

### 1.5 The present study

The aim of the present study is to understand whether mistrust and conspiracy beliefs undermine people's vaccination intention in Austria. We hypothesised that those who hold more mistrust in governmental organizations and inclination to support conspiracy theories related to COVID‐19 tend to harbour lower intentions to receive a COVID‐19 vaccine. We used the TRA as the psychological model to examine the prediction of the vaccination decision at a preimplementation stage of a vaccine rollout. It was hypothesised that the TRA constructs (i.e., attitude, subjective norm) would predict the vaccination intention regardless of one's level of mistrust and conspiracy and that a more favourable attitude and more perceived pressure from important others would be associated with people's stronger vaccination intentions.

Our final research aim was to profile the cluster members by comparing several variables that may play a role in shaping one's vaccination scepticism or hesitancy. Previous research suggests that general vaccination confidence (Kalimeri et al., 2019), populist political inclination (Peretti‐Watel et al., 2020), trust in political institutions (Kennedy, 2020), and exposure to conspiracy beliefs (Goldberg & Richey, 2020; Jolley & Douglas, 2014; Romer & Jamieson, 2020) could be the underlying reasons of the low trust in vaccination. People who perceive their own health status as good are less likely to become vaccinated (World Health Organization, 2016). Thus, the between‐cluster differences on these variables will be explored in the Austrian sample.

## 2 METHODS

### 2.1 Participants and procedure

A cross‐sectional online questionnaire was developed and then circulated via the university's email system in August 2020. The target group was anyone over the age of 18 years and the recipients of the study invitation were currently enrolled university students. After having read information regarding the research project, participants gave their consent and then proceeded to the survey. A debriefing procedure was carried out after the final question. Participants were able to quit the questionnaire at any point without negative consequences. None of the questions was mandatory, but participants had to actively select “I do not want to answer that question” if they wanted to skip a specific item. The study's procedure and questions underwent ethical consideration and were approved by the university's ethics committee. There were 217 participants who took part in the study (female 80.4%; *M*_age_ = 22.98 years; 31.9% had completed tertiary education).

### 2.2 Measures

The questionnaire assessed demographic data (i.e., gender, age, highest educational level, country of residence) and the following variables. The TRA constructs were based on established guidelines (Ajzen, 1991) and validated items from previous studies. All scales showed good internal consistencies and the mean score was used to represent the construct. The survey was developed in English and then translated into German.

#### 2.2.1 Vaccination intention

Adapted from a previous study on swine flu vaccine (Myers & Goodwin, 2011), intention was assessed with three questions (i.e., *Do you intend to get yourself vaccinated against COVID‐19?; I would try to get vaccinated as soon as possible; I would participate in vaccine‐tests before the vaccine is approved; α* = 0.82) using a 7‐point Likert scale ([1] *definitely not* to [7] *definitely yes*). Higher scores reflect a stronger intention to receive the COVID‐19 vaccine.

#### 2.2.2 Attitude

Six semantic differential items were used on a 7‐point scale (e.g., [1] *wise* to [7] *foolish; α* = 0.97), as in a previous vaccine study (Myers & Goodwin, 2011). Some items were reverse coded so that higher scores reflect a positive attitude toward having the COVID‐19 vaccine.

#### 2.2.3 Subjective norm

Four items were used to examine subjective norm (e.g., *Those people who are important to me would want me to receive the vaccine for COVID‐19; α* = 0.97), on a 7‐point Likert scale ([1] *strongly disagree* to [7] *strongly agree*). These items are adapted from a previous study on health behaviour (Zhao, White, et al., 2019). Higher scores indicate higher perceived pressure one feels from significant others to have the COVID‐19 vaccine.

#### 2.2.4 Political self‐placement

This variable was measured on a far‐left to far‐right continuum with a 10‐point scale (Breyer, 2015). This scale uses ten letters (i.e., F, A, M, O, G, Z, E, Y, I, P) as choices to avoid centralised answers; the letters were subsequently converted to scores. Higher scores reflect a right‐wing political position, whereas lower scores indicate a left‐wing position. This item has been used in the annual ALLBUS (German General Social Survey) since the 1960s. With the 10‐point scale, a tendency to the centre is avoided. The measure was considered appropriate for our study as an established, well validated, and time‐efficient measure.

#### 2.2.5 Trust

A validated nine‐item scale (e.g., *When it concerns handling the COVID‐19 pandemic, the government acts in the interest of its citizens*; α = 0.95) was used to assess trust in governmental organisations in relation to COVID‐19 ([1] *totally disagree* to [7] *totally agree*), adapted from a previous study (Grimmelikhuijsen & Knies, 2017). Higher scores show more trust in the government's actions on COVID‐19.

#### 2.2.6 Conspiracy ideation

A single‐item measure (i.e., *I think that the official version of the events given by the authorities very often hides the truth*) was used to assess the conspiracy ideation ([1] *completely false* to [9] *completely true;* Lantian et al. (2016)). Higher scores reflect higher levels of conspiracy ideation. This item assesses a general conspiracy inclination and was ideal for use in the present study as we did not want to investigate COVID‐19 specific conspiracy beliefs. This item is well‐validated and shows a high stability over time as well as good psychometric qualities (Lantian et al., 2016) and has been used in previous COVID‐19‐related research (e.g., Hornsey et al., 2021; Sutton & Douglas, in press).

#### 2.2.7 Confidence in vaccination

Confidence in vaccinations was assessed with 3 items (*α* = 0.92) based on the validated vaccination confidence scale (Betsch et al., 2018). The items (e.g., *I am completely confident that vaccines are safe*.) were measured using a 7‐point Likert scale ([1] *totally disagree* to [7] *totally agree*). Higher total scores reflect more confidence in vaccine effectiveness and the government's management of the pandemic. This scale has been previously used in COVID‐19‐associated studies (e.g., Luo et al., in press; Solís Arce et al., 2021).

#### 2.2.8 Self‐perceived health status

Self‐perceived health was measured with a single item (i.e., *In general, how do you consider your overall physical health?*; Subramanian et al. (2010)), on a 5‐point scale ([1] *very good* to [5] *very poor*). Items were reverse coded so that higher scores reflect a better self‐perceived health status. This item has been used extensively in previous research as a measure of self‐reported health status and is taken from the 2002 World Health Survey carried out by the World Health Organization (Subramanian et al., 2010).

### 2.3 Analysis

Given the exploratory nature of this study, descriptive analyses were first conducted to examine the correlational relationships among the variables. The two‐step cluster analysis was adopted to identify the heterogenous clusters based on trust and conspiracy ideation. As its name suggested, this approach of cluster analysis includes two steps, namely, to separate groups using a distance measure (e.g., log‐likelihood) and to select the optimal cluster model based on probabilistic methods (e.g., Bayesian information criterion) (Gelbard et al., 2007). The two‐step cluster analysis has several advantages compared with traditional cluster analysis approaches and is regarded as a reliable subgroup detecting method (Benassi et al., 2020; Kent et al., 2014). Based on the identified clusters, the COVID‐19 related variables and demographic variables were compared between clusters. Finally, following the TRA, regression analyses were used to examine the relationships between intention and its antecedents (i.e., attitude, subjective norm) by cluster. Given the nonnormal distribution of intention, robust estimator was applied. A Wald test was employed to examine the intervariable invariance across clusters. IBM SPSS 26 and Stata 15.1 were used for the data analyses.

## 3 RESULTS

### 3.1 Descriptive findings

Tables 1 and 2 summarise the descriptive findings of study variables. Notably, although the mean of vaccination intention was 3.02 (i.e., just below the midpoint of the scale), there were 43 participants (22.4%) who reported the lowest level of intention (i.e., the smallest value on the scale). Table 2 shows that intention had a strong association with attitude, subjective norm, and confidence in vaccination. Intention also had moderate correlations with trust and conspiracy. The association between intention and education suggests participants who had tertiary education showed a stronger intention to receive a COVID‐19 vaccine. Higher education was also related to stronger subjective norm and lower conspiracy beliefs.

**Table 1:** Descriptive findings among all participants and cluster comparisons

Variable	All	Reference cluster	Sceptics cluster
	(*N* = 217)	(*N* = 89)	(*N* = 104)
Female (%)	80.4%	70.9%	85.6%
Age (years), mean (SD)	22.98 (22.34)	24.60 (21.08)	20.77 (25.38)
Tertiary education (%)	31.9%	40.9%	24.0%
Intention to receive COVID‐19 vaccine	3.02 (1.68)	3.83 (1.66)	2.39 (1.42)
Positive attitude toward COVID‐19 vaccine	4.90 (1.88)	5.73 (1.40)	4.19 (1.97)
Subjective norm of receiving COVID‐19 vaccine	4.26 (1.80)	4.85 (1.63)	3.75 (1.80)
General confidence in vaccination	4.65 (1.70)	5.48 (1.44)	3.93 (1.63)
Trust in government's COVID‐19 measures	2.90 (1.02)	3.33 (0.98)	2.54 (0.89)
Conspiracy	5.15 (2.42)	2.87 (1.16)	7.12 (1.17)
Political attitude (right self‐placement)	4.29 (1.69)	3.99 (1.77)	4.56 (1.59)
Self‐perceived health status	4.32 (0.72)	4.34 (0.64)	4.28 (0.80)

*Note*: One nonbinary gender participant was identified, but not included in analyses.

**Table 2:** Pearson correlation among study variables

	1	2	3	4	5	6	7	8	9	10	11
1. Intention	‐										
2. Positive attitude	0.83***	‐									
3. Subjective norm	0.81***	0.80***	‐								
4. Far‐left to far‐right continuum	−0.12	−0.09	−0.07	‐							
5. Trust	0.56***	0.53***	0.51***	0.04	‐						
6. Conspiracy	−0.46***	−0.50***	−0.37**	0.23**	−0.40***	‐					
7. Confidence in vaccination	0.73***	0.81***	0.69***	−0.10	0.60***	−0.53***	‐				
8. Perceived health	0.06	0.10	0.09	0.09	0.09	−0.03	0.08	‐			
9. Gender	−0.02	0.08	0.05	0.14	−0.00	−0.18*	0.15*	0.06	‐		
10. Age	0.02	0.06	0.07	−0.23**	0.07	−0.11	0.05	0.01	0.06	‐	
11. Tertiary education	0.15*	0.11	0.15*	−0.04	0.06	−0.20**	0.10	−0.04	0.16*	0.12	‐

*Note*: Gender: 1 = Male, 0 = Female.

* *p* < .05

** *p* < .01

*** *p* < .001.

### 3.2 Identifying two clusters

Based on trust in the government's COVID‐19 response and endorsement of COVID‐19 conspiracy beliefs, two clusters were identified using the two‐step cluster method. The final cluster solution showed a good degree of cohesion and separation (Average Silhouette = 0.6). Given the profiles, these two clusters were called “reference cluster” (i.e., with more trust in government's COVID‐19 measures and lower conspiracy beliefs) and “sceptics cluster” (i.e., with less trust in the government's COVID‐19 measures and greater conspiracy beliefs).

Between‐cluster comparisons (Table 1) showed that, apart from perceived health (*F*_(1, 188)_ = 0.35, *p* = 0.553), all other COVID‐19 variables had significant differences between clusters. Specifically, the sceptics cluster members had lower intentions to receive a COVID‐19 vaccine (*F*_(1, 183)_ = 40.26, *p* < 0.001), less favourable attitudes (*F*_(1, 184)_ = 35.93, *p* < 0.001), lower subjective norm (*F*_(1, 183)_ = 18.78, *p* < 0.001), lower confidence in general vaccination (*F*_(1, 191)_ = 48.24, *p* < 0.001), less trust in the government's COVID‐19 response (*F*_(1, 191)_ = 34.14, *p* < 0.001), greater beliefs in COVID‐19 conspiracy theories (*F*_(1, 191)_ = 633.99, *p* < 0.001), and were more toward the right in political views (*F*_(1, 179)_ = 5.27, *p* = 0.024). As for the demographic variables, there was no associations between cluster membership and age (*F*_(1, 191)_ = 1.272, *p* = 0.261), but more women (*χ* ^2^_(1)_ = 6.08, *p* = 0.014) and fewer tertiary‐educated participants (*χ* ^2^_(1)_ = 6.26, *p* = 0.012) were found in the sceptics cluster.

### 3.3 Inter‐variable relationships

Following the TRA, model results from robust regression showed that attitude and subjective norm were significant predictors of intention in both clusters (Table 3). While the subjective norm‐intention link had no significant cross‐cluster difference, the attitude‐intention link was weaker in the sceptics cluster (*χ* ^2^_(1)_ = 8.13, *p* = 0.004).

**Table 3:** Robust regression predicting intention to receive COVID‐19 vaccination by cluster

	Reference cluster		Sceptics cluster		Wald test
	(*N* = 89)		(*N* = 102)		
	*B*	*p*	*B*	*p*	
Positive attitude	0.57	<0.001	0.51	<0.001	*χ* ^2^_(1)_ = 8.13, *p* = 0.004
Subjective norm	0.37	<0.001	0.39	<0.001	*χ* ^2^_(1)_ = 0.39, *p* = 0.531

*Note: B* = standardised coefficient.

## 4 DISCUSSION

With COVID‐19 continuing to batter the world, vaccination appears to be an effective prevention. Based on a sample of predominantly younger, educated women from Austria, we used cluster analysis to extract a cluster who likely possessed more mistrust of government responses to COVID‐19 management and inclination to support COVID‐19 conspiracy theories. The cluster members showed significantly lower scores on pro‐vaccine perceptions than the reference cluster members. As hypothesised, vaccination intention could be predicted by one's attitude and subjective norm, consistent with the TRA. The identification of the cluster with weaker intention to be vaccinated and their profiles provide important implications for the health communication for the impending COVID‐19 vaccination.

Of concern, 22.4% of the participants reported the lowest score on the intention scale to have a COVID‐19 vaccine. While this status mirrors the low vaccination willingness among Europeans (Larson et al., 2016), this reluctancy may add uncertainties to the COVID‐19 situation in Europe. Consistent with previous findings (Graffigna et al., 2020; Hornsey et al., 2018), age and gender were unrelated to vaccination intention in our sample. However, having completed tertiary education was related to higher vaccination intention and less support for COVID‐19 conspiracy theories, consistent with an American study (Goldberg & Richey, 2020). By contrast, the two constructs from the TRA—attitude and subjective norm—showed the largest correlations with vaccine intention, followed by confidence in general vaccination. Both attitude and subjective norm were significant predictors of vaccination intention. These findings support the utility of the TRA in understanding people's vaccination intention. Interestingly, the difference for the attitude‐intention link between clusters suggests that positive attitudes toward vaccination intention may be more difficult to translate to vaccination intention if people harbour less trust in the government management of COVID‐19 and more conspiracy theories.

The existence of clusters based on trust and conspiracy theories further explains some underlying reasons that may influence people's decision making for vaccination. Notably, the sceptics cluster members tend to hold a politically right position. This association between politically right‐wing and lower vaccination intention was also identified in previous European studies (Engin & Vezzoni, 2020; Ward et al., 2020). A growing body of recent studies found that people with higher conspiracy ideation are unlikely to obey the medical recommendations (instead, they tend to refuse vaccination and support hydroxychloroquine) (Bertin et al., 2020; Roozenbeek et al., 2020), like our findings in Austria. Consistent with previous research (Goldberg & Richey, 2020), our study shows that trust in government is protective against conspiracy beliefs.

The clusters do not imply a fixed stereotype as the conspiracy about vaccination could be rooted in concrete factors such as negative emotions (Tomljenovic et al., 2020) and even the amount of exposure to mainstream media (Romer & Jamieson, 2020). Our study suggests that increasing people's positive attitude toward COVID‐19 vaccination and establishing a provaccination social norm could theoretically improve the vaccination intention. However, as indicated by the cluster profiling, changes in beliefs and behaviours may be difficult as political position and conspiracy ideation are not easy to be influenced (Taylor, 2019). For a pandemic such as COVID‐19, promoting its vaccines may have to involve abolishing the pure biomedical information‐based education approach; instead, health communication should target culturally and politically infused backgrounds as discussed for other health behaviours (Lyons & Chamberlain, 2017; Zhao, Young, et al., 2019). As far as current strategies in Austria are concerned, official vaccine‐promoting guidelines merely centre on information delivery from medical professionals (e.g., information about the vaccine safety; Bundeskanzleramt Österreich, 2020). Based on our findings, different angles targeting political influences and general concerns about vaccines are needed. Social marketing strategies may also be used to influence the public, as one effective way to combat conspiracy ideation is to create a stronger mindset against the conspiracy theories (Taylor, 2019).

Despite the contribution of the study providing further understanding of the pertinent influences on people's vaccine intentions in the preimplementation stage of a vaccination rollout, this study has some limitations. First, the participants are limited to well‐educated Austrians, mostly women. Nevertheless, if these findings have emerged in a predominantly educated sample, the identified differences between the clusters in our sample may be more pronounced in the general population given previous findings suggesting conspiracy theories are endorsed more in people with lower education levels (Biasio et al., 2021; Duplaga, 2020). Second, although the majority of scales employed in the present study used established multi‐item measures, single‐item scales were used for a few of the constructs. Although the single‐item scales were based on previously established and/or validated measures, their psychometric property may be enhanced with more items especially with scales that were originally developed in non‐Austrian contexts. Third, with the increasing availability of COVID‐19 vaccines, examining perceptions of control (e.g., control beliefs) is needed where models such as the Theory of Planned Behaviour (Ajzen, 1991) may be more applicable. In addition, as vaccination hesitancy is multifactorial, a broader theoretical perspective is also needed in future research to capture the contextualised meanings of this hesitancy. Finally, the timing of the study precluded the collection of follow‐up data to enable linking of identified predictors and intentions with people's vaccination decisions. Nevertheless, we believe it remains important to gauge people's intentions during the preliminary stages of vaccine implementation to understand the pertinent predictors of vaccination willingness and resistance given their broad indication of people's likely subsequent vaccination decisions. Overall, our research provides important avenues for policy makers and health interventionists to understand vaccination intentions during the COVID‐19 pandemic, especially during their preimplementation stage. Future campaigns based on the continuing development and release of new COVID‐19 vaccines and booster shots may need to consider political background in the community for a more stratified approach.

## CONFLICT OF INTERESTS

The authors declare that there are no conflict of interests.

### PEER REVIEW

The peer review history for this article is available at https://publons.com/publon/10.1002/jcop.22714

## Data Availability

The data that support the findings of this study are available from the corresponding author upon reasonable request.
